# The Interplay Between Neuromodulation and Stem Cell Therapy for Sensory-Motor Neuroplasticity After Spinal Cord Injury: A Perspective View

**DOI:** 10.3390/jcm15020879

**Published:** 2026-01-21

**Authors:** Anthony Yousak, Kaci Ann Jose, Ashraf S. Gorgey

**Affiliations:** 1 Department of Biology, School of Science, Indiana University Indianapolis, Indianapolis, IN 46202, USA; 2 School of Medicine, Virginia Commonwealth University, Richmond, VA 23298, USA; 3 Spinal Cord Injury and Disorders Center, Richmond VA Medical Center, Spinal Cord Injury & Disorders Service, 1201 Broad Rock Blvd, Richmond, VA 23249, USA; 4 Department of Physical Medicine and Rehabilitation, Virginia Commonwealth University, Richmond, VA 23298, USA

**Keywords:** spinal cord epidural stimulation, neuromodulation, neuroplasticity, SCI, task-specific training, stem cell therapy, mesenchymal stem cell therapy

## Abstract

Spinal Cord Injury (SCI) rehabilitation is undergoing a transformative shift with the emergence of new treatment strategies. Historically, treatment options were limited, and few offered meaningful recovery. Recent work in human models has shown that neuromodulation specifically with spinal cord epidural stimulation (SCES) paired with task-specific training (TsT) can partially restore motor function such as the ability to stand, step, and perform volitional movements. Despite these advances, the recovery has been shown to plateau even with the combination of therapies. The recovery process typically leads to partial rather than complete restoration of function. This limitation arises because current approaches primarily reactivate existing circuits rather than repair the disrupted pathways. Scar tissue and loss of descending and ascending connections remain major barriers to full recovery, restricting the transmission of neural signals. We argue that the next phase of research should be a synergistic strategy building upon the successes of neuromodulation and TsT while incorporating a regenerative therapy such as stem-cell-based interventions. Whereas neuromodulation and task-specific training increases excitability and reorganizes existing networks, stem cells have the potential to repair structural damage and re-establish communication across injured regions or facilitating the establishment of dormant pathways. The future of SCI recovery relies on multi-modal synergistic interventions that are likely to maximize long-term functional outcomes. In the current perspective, we summarized the basic findings on applications of SCES on restoration of sensory-motor functions. We then projected on current interventions on utilizing stem cell therapy intervention. We highlighted the outcomes of randomized clinical trials, and the major barriers for considering the synergistic approach between SCES and stem cell intervention. We are hopeful that this perspective may lead to roundtable scientific discussion to bridge the gap on how to conduct numerous clinical trials in the field.

## 1. Introduction

Spinal Cord Injury (SCI) is a devastating medical condition resulting in a detrimental impact on a person’s quality of life [1]. In the United States, approximately 305,000 individuals are affected by SCI, with an estimated 18,000 new cases occurring annually [1]. The financial burden of SCI varies depending on age and severity, with lifetime costs starting at approximately $1.4 million [1]. Based on the level of injury, SCI interrupts sensory-motor and autonomic pathways, resulting in complete and incomplete paralysis [2]. Major causes of traumatic SCI are motor vehicle accidents, sports, and violence [1,3]. Non-traumatic SCI can be caused by degenerative disorders, vascular insults, neoplasms, inflammatory diseases, and infections [4].

Beyond the primary concern of motor impairment, injury above T6 is accompanied with dysfunction in the autonomic nervous system [5]. There are also complications with the parasympathetic nervous system; however, this paper is geared towards improvement in the somatosensory nervous system. Dysfunction in autonomic nervous system may impact several vital systems including thermoregulatory and cardiovascular systems, loss of bladder, bowel, and sexual functions [6], which is beyond the scope of the current work [1].

Following trauma, the primary injury occurs in the form of physical damage, such as compression, distraction or laceration of the spinal cord [2,7]. This is followed within 24–72 h with cascades of secondary damage to neural and axonal structures of the cord [8]. This is accompanied by inflammatory cascades that ultimately cause neuronal apoptosis as well as leukocytosis and further damaging the axons at the site of trauma as well as the bordering segments of the spinal cord [8,9]. Preclinical studies have shown that early anti-inflammatory intervention, such as minocycline administration can limit lesion expansion following acute SCI [10]. However, these findings are largely preclinical, and further studies need to be established before clinical relevance can be established. The final stage of the secondary injury is arachnoiditis with factors that inhibit axonal growth [2]. Secondary injury is characterized by vascular dysfunction, neuroinflammation, cascades of oxidative stress and different immune-macrophage reactions [11]. This results in cell death and damage to neural networks and subsequently loss in sensory, motor and autonomic functions [11]. The process of secondary injury can persist for a few days or several years following SCI, forming both physical and chemical roadblocks to axonal generation [9]. Today, there is no established treatment to manage SCI and facilitate restoration of somatosensory control, which is the ability of the intervention to increase sensory score and motor scores as determined by the ASIA impairment scale [12]. This resulted in several medical comorbidities, long-term costly consequences and impaired quality of life [1]. Several of these comorbidities have been well studied and highlighted in previous work [2,11,12].

### 1.1. Neuroplasticity

Neuroplasticity is the central nervous system’s (CNS) ability to establish new neurons or synaptic connections in response to motor learning and stimulation [7,13]. This is not limited to restoration of intraspinal pathways but also to changes in functional connectivity throughout the entire central nervous system, including supraspinal areas and the cortex [8,14]. SCI, after the primary injury, can mechanically impact neural tissues and axons by severing connections and rupturing blood vessels [2]. Moving into secondary injuries and scar tissue formation, the essential neural circuits are disrupted and the descending supraspinal control is lost [2,9]. Neuroplasticity has been researched to promote the development of segmental and spinal pathways, potentially restoring connectivity in the affected areas [13,15]. Sensory-motor neuroplasticity is a specific subcategory that is important to SCI recovery and is the focus of the current perspective. Understanding the mechanism underlying neuroplasticity and establishing reliable methods for measuring its progression is essential for developing effective treatment strategies.

The two fundamental interventions that may influence neuroplasticity in SCI rehabilitation are neuromodulation and TsT [16]. Neuromodulation is a therapeutic technique which aims to enhance nervous system activity through target stimulation [17]. Neuromodulation works by delivering an external stimulus to modulate the excitability and communication between neurons [18]. External stimulation can be induced either chemically or electrically. Chemical stimulation typically refers to serotonergic drugs that modulate neural activity to promote functional recovery [17]. In the case of neuroplasticity in recovering SCI, electrical stimulation is the primary source of neuromodulation [18]. This involves delivering target stimulation to specific parts of the nervous system, such as the lumbosacral segments, to reactivate the neural pathways and influence reorganization of the disrupted pathways after SCI [18]. These neural pathways involve posterior large afferent fibers, proprioceptive spinal fibers, segmental interneurons, motor efferent fibers and either ascending or descending tracts. Research is underway to highlight the significant contribution of each pathway to the process of neuromodulation after SCI.

Task-Specific Training (TsT) is the second fundamental component that enhanced neuroplasticity after SCI. Task-specific training has been defined as the restoration of a specific motor behavior through a highly repetitive intensive approach that engages both sensory and afferent feedback to induce specific motor functions like standing or stepping [12,16]. It is a rehabilitation technique which focuses on repetitive performance of a goal-directed movement to facilitate functional recovery [12,16]. Examples of a goal-directed movement may include body-weight-assisted treadmill walking, robotic gait training, or most movements that help improve motor learning and promote reorganization of neural pathways [12]. By engaging the neural circuits in specific movements, it strengthens the connection between sensory and motor training to restore functional movements [16]. In the context of SCI, TsT is particularly effective when paired with neuromodulation [15,16]. The stimulation of neural networks from internal and external stimulation has been shown to enhance synaptic plasticity [15]. Although neuroplasticity can lead to recovery after SCI, there are many factors that may disrupt this process, and the effectiveness of synergistic interventions may vary [14]. For example, epidural stimulation outcomes vary with injury severity, stimulation parameters, and TsT, which can differentially enhance or impair the recovery of a person after SCI [14]. Therefore, neuroplasticity is not uniformly beneficial and when combined with neuromodulation and TsT, it may cause some maladaptive plasticity that limits functional recovery and contributes to the development of neuropathic pain or spasticity [19].

### 1.2. Neuromodulation

**Figure 1:**
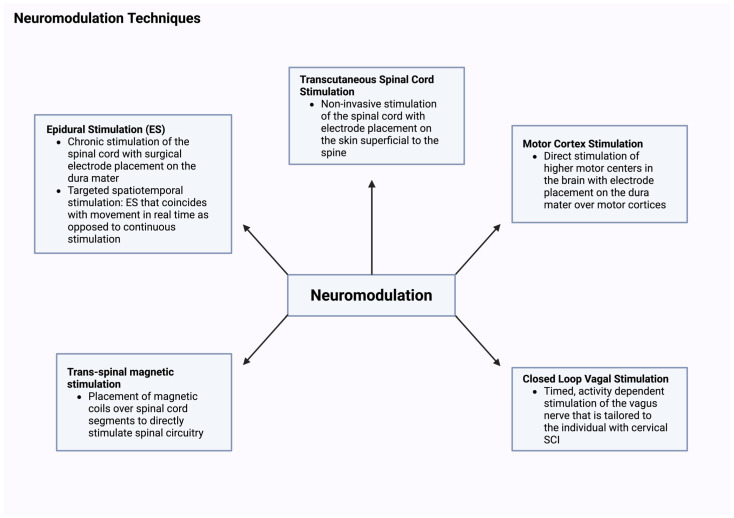
Pictorial diagram of some commonly used neuromodulation therapies [14,25,26,27,28,29,30,31,32,33,34,35,36] that are currently explored. Created in BioRender web application. Jose, K. (2026) https://BioRender.com/xc03jsb, accessed on 19 January 2026.

Despite challenges, a new era has emerged with promising hope for restoration of sensorimotor control in persons with SCI [17,18,20]. Two decades of research highlighted the significance of using several neuromodulation interventions in conversion of the dormant lumbosacral segments into more active tonic and rhythmic circuitries that enhance standing and stepping after SCI [20]. Previous work highlighted the capability of lumbosacral central pattern generator to independently function without supraspinal control [21]. This has led researchers to execute a number of research studies that effectively prove that the spinal cord can act independently form the cortical supraspinal control and be characterized as a smart system [14]. Repetitions of specific tasks have led to the development of motor control behavior upon eliciting external stimuli [18,20]. The spinal cord can transform streams of sensory information into effective motor behavior (i.e., smart cord) as well as dampen unnecessary reflex behaviors that may lead to muscle spasms or spasticity [17,18]. The theory and applications of central pattern generator or spinal cord locomotor centers have been well studied over the years [6,18,22]. These studies were guided by almost three decades of animal research that examined neuroplasticity following decerebration and SCI models. Therefore, providing the most effective triggering stimuli is key for unlocking spinal cord activities into more translational behavior [6]. Furthermore, beyond enabling central pattern generators, epidural stimulation facilitates transmission from supraspinal centers to regions below the level of injury [23,24]. This is enhanced by increasing the excitability of the supraspinal center which allows for the weak or latent stimulation to be able to be transmitted across the lesion [23,24]. Other experimentally approved neuromodulation approaches may operate at the cortical, subcortical or spinal level to enhance motor function in persons with neurological disorders. One approach is vagus nerve stimulation, which can be administered either invasively or noninvasively, and works effectively when paired with TsT [25,26,27]. It is used to treat not only SCI but has been used for rehabilitation after stroke [26,27]. Figure 1 below depicts various appr neuromodulation strategies that are currently explored at different phases of clinical trials.

**Figure 2:**
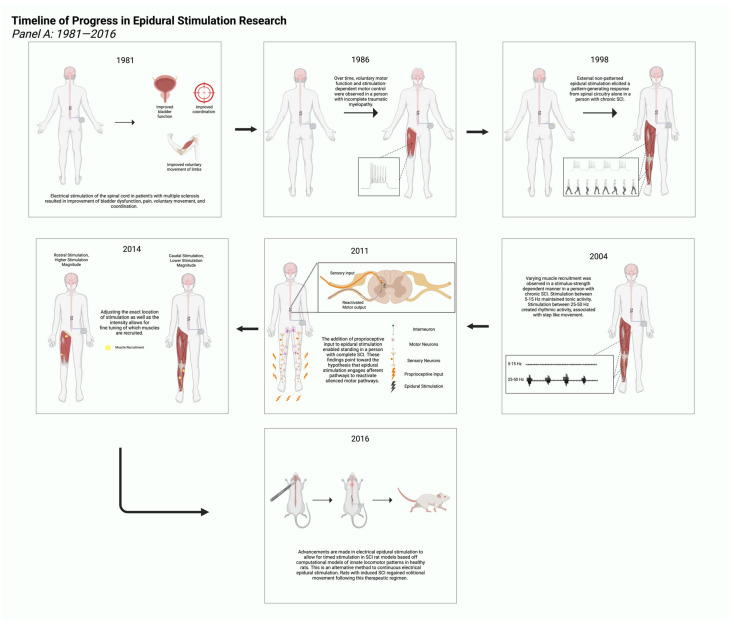

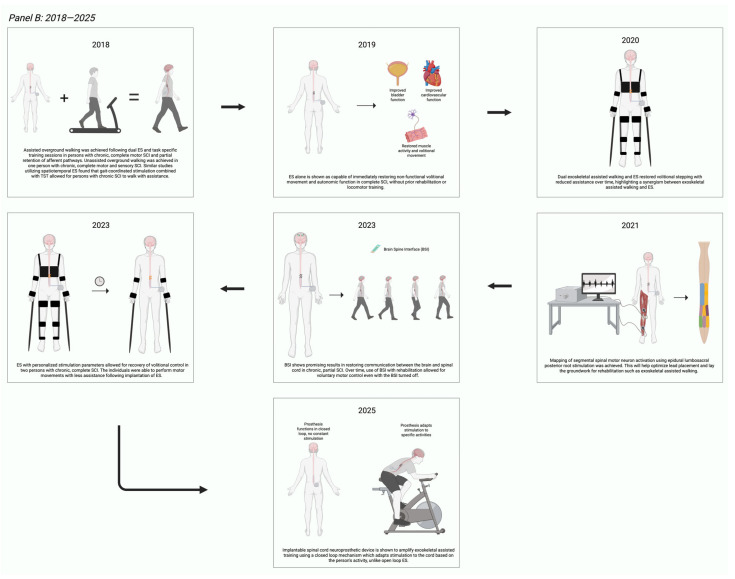
Milestones in epidural stimulation research spanning the last 39 years. Advancements in research have unraveled the potential for epidural stimulation to guide modern therapies for SCI recovery [5,6,14,21,22,28,29,35,39,40,41,42,43,44,45,46]. Panels (**A**,**B**) highlight representative research findings from 1981 to 2016 and 2018 to 2025, respectively. Created in BioRender web application. Jose, K. (2026) https://BioRender.com/2l0rw1f, accessed on 19 January 2026.

Combination of neuromodulation and TsT has been shown to be an effective rehabilitation strategy in enhancing locomotor behaviors after SCI [6,28,29]. It is still not entirely clear how such mechanistic synergism between electrical neuromodulation and TsT may work [19]. Neuromodulation delivers electrical impulses to the lumbosacral segments to unlock the spinal cord locomotor centers to either trigger a tonic response or a rhythmic oscillatory response to initiate motor function like standing and stepping [14,19]. TsT in the form of body-weight-supported treadmill training has been used intensively to demonstrate the ability of the spinal cord to induce rhythmic stepping that is later translated into overground stepping with or without assistive devices [6,28,29]. Dual application of neuromodulation and TsT has been shown to have an amplified effect on restoration of volitional movement in persons with SCI [37]. Another effective TsT tool is the application of robotic exoskeletons as an effective neurotherapeutic approach to restore motor control after SCI [38,39,40,41]. Exoskeletal-assisted walking (EAW) was effectively used in the rehabilitation of persons with SCI [38]. The initial studies were geared towards improvement of the level of physical activity and quality of life in persons with SCI [38]. EAW could enhance overground locomotion and step into a variety of injuries [38]. EAW is characterized by low metabolic cost which is likely to be used as an effective therapeutic intervention to facilitate repetitions of movements without ensuing fatigue in persons with SCI [38,39,40]. EAW was used safely with spinal cord epidural stimulation (SCES) to facilitate active stepping in a fashion that enhances motor recovery as measured by EMG after SCI [39]. Figure 2 highlights milestones in epidural stimulation research between 1986 and 2025.

#### 1.2.1. Neuromodulation in the Animal Model

Due to the limitations associated with recruitment of persons with SCI, the use of rat models is a promising first step in identifying potential preclinical therapeutic strategies for management of participants with SCI [8,20,23,24,31,47]. Animal models have been used to study the efficacy of neuromodulation therapy alone on enhancing neuroplasticity in both uninjured rats and those with SCI [8,20,31]. In Table 1, we summarize a number of studies that determined the effectiveness of dual neuromodulation and TsT in the animal model.

A review of studies using animal models ultimately displays that stimulation of neuronal activity through neuromodulation can promote generation of new neural pathways [20,23,24]. The findings of the research showed the epidural stimulation acutely raised spinal excitability. Paired with TsT, it strengthens synaptic connectivity within spared circuits [20]. Repeated stimulation produces measurable plasticity which can be seen with improved stepping/weight control or with EMG readings [21].

Although neuromodulation and TsT have been well studied over the past few decades, Sharif et al. 2021 explains a key point that the degree to which descending neural pathways are injured impacts whether TsT alone will show significant improvement in SCI [8]. Therefore, due to the near-complete damage of descending pathways in complete SCI, it is unlikely that rehabilitation alone can restore motor function [8]. They hypothesized that to regain motor function, neuroplasticity in both descending and afferent neural fibers is necessary. Specifically, researchers propose that there must be a decrease in proprioceptive afferent (PA) fibers and an increase in corticospinal tract (CST) fiber regeneration [8]. In this model, regeneration and strengthening CST connections must be accompanied by a relative reduction in excessive PA influence on spinal interneurons, this is because PA competitively occupies synaptic space when descending inputs are weakened [8]. This is consistent with symptoms of hyperreflexia in individuals with SCI [8]. Sharif et al. 2021 present results that are consistent with a decrease in PA fiber density in the synaptic space with synergistic neuromodulation and rehabilitation when compared to rehabilitation alone [8]. This is consistent with their hypothesis because combining neuromodulation with TsT reduced markers of PA fiber dominance within the spinal cord, which favored re-engagement of CST pathways compared to rehabilitation alone [8]. These preclinical trials with animal models have laid the foundation for translating these results into human clinical trials, which can be seen in Table 2.

#### 1.2.2. Neuromodulation in the Human Model

Table 2 outlines human studies and provides evidence to demonstrate the mechanisms through which neuromodulation and TsT facilitate neuroplasticity in SCI rehabilitation. Neuromodulation primarily alters the excitability of spinal networks, allowing supraspinal inputs to regain influence over motor circuits. For example, Rejc et al. showed that a person with chronic, motor-complete SCI regained volitional lower-limb movement and independent standing following long-term activity training with lumbosacral epidural stimulation (ES) [33]. Similarly, Harkema et al. demonstrated that with continued ES, participants exhibited voluntary control of ankle, knee, and toe movements, as well as task-specific EMG activity during standing and stepping [14]. These findings suggest that neuromodulation promotes the reactivation of functionally silent pathways and increases motor neuron excitability.

In a randomized controlled trial, Comino-Suarez et al. (2025) found that participants undergoing robotically assisted walking with transcutaneous SCS (tSCS) significantly improved their Lower Extremity Motor Scores, walking speed, and functional mobility, compared to those who received sham stimulation [30]. Similarly, Mckenzie et al. reported that combining gait training with acute intermittent hypoxia and tSCS led to greater improvement in the Timed Up and Go test than either intervention alone, indicating that repetitive motor practice strengthens sensorimotor integration and improves functional mobility over time [16]. Human studies are accompanied with extensive spinal mapping that determines the exact stimulation parameters and configurations necessary to activate the spinal cord [42,43,44]. The process is rather complex when implanted brain–computer interface is bridged to control that implanted epidural stimulation in a phase-dependent manner [44]. Furthermore, closed-loop spinal stimulation successfully enhanced motor outcomes and ensured functional recovery in persons with SCI [41,48].

While each intervention contributes towards neuroplasticity independently, combined neuromodulation and TsT appear to drive neuroplasticity further than either method alone. Kazim et al. provided evidence of CST sprouting and widespread reorganization throughout the motor system following paired ES and TsT, which can be seen in the table above about human research [34]. Review of animal and human studies share a similar emerging hypothesis that neuromodulation primes the nervous system, while TsT shapes the functional circuits. Although this combinatory approach has shown partial recovery of motor function in some cases, our goal is to identify the optimal combination of interventions to maximize recovery of motor control.

### 1.3. Stem Cells

**Figure 3:**
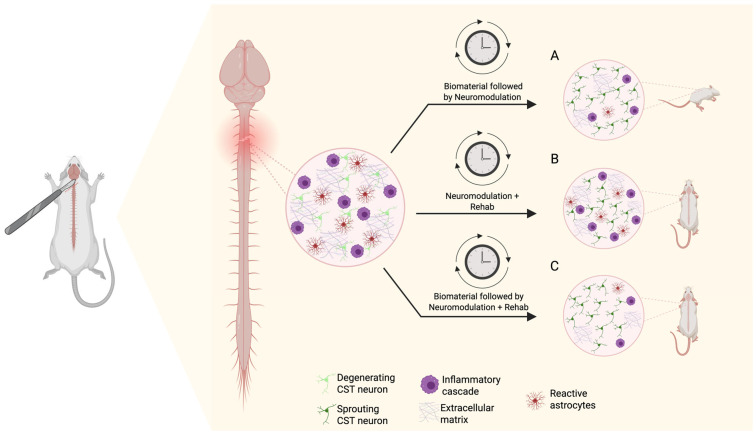
(**A**) Injection of healing biomaterial 3 days after induced SCI in rats followed by neuromodulation therapy allowed for significant CST sprouting, reduced inflammation, and restricted scar tissue formation [8]. (**B**) Neuromodulation combined with task-specific rehabilitation allowed for synergistic fine tuning of regenerated CST axons, allowing injured rats to regain motor function to a greater degree than each therapy could alone [8]. (**C**) We propose that injection of biomaterial prior to dual neuromodulation and task-specific training would create an even more robust regeneration of fine motor function in rats with SCI. Created in BioRender web application. Jose, K. (2026) https://BioRender.com/brh0iv6, accessed on 19 January 2026.

**Figure 4:**
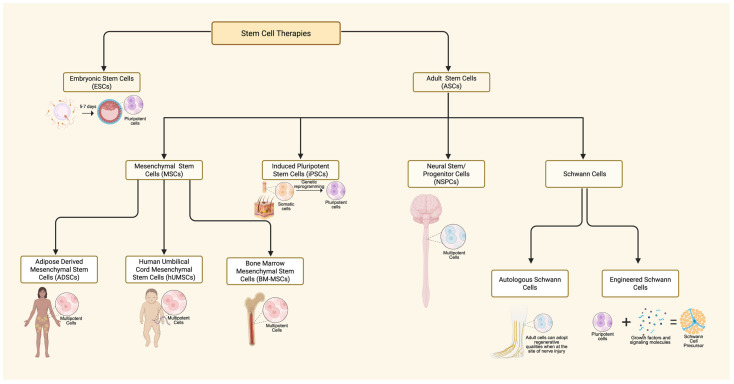
Flow chart of various forms of stem cell therapies used in clinical trials for SCI treatment as well as their origins [50,51,55,56]. Created in BioRender web application. Jose, K. (2026) https://BioRender.com/bs0exly, accessed on 19 January 2026.

We highlight the potential effects of stem cell therapy as a third rehabilitation avenue in addition to neuromodulation and TsT for SCI recovery. Figure 3 highlights a graphical presentation based on animal research that demonstrates the synergistic application of stem cell therapy in combination with neuromodulation. Stem cell therapy is an experimental treatment option that offers an optimistic future for recovery following severe SCI through regenerative medicine. Mesenchymal stem cells (MSCs) hold a lot of potential due to their multipotency as well as their availability in various mature tissues such as bone marrow, adipose, endometrium, and others [49]. There are various forms of MSC harvesting, a few commonly used ones being bone-marrow-derived stem cells, adipose-derived stem cells, as well as human umbilical-cord-derived stem cells [50,51]. Adipose-derived stem cells (ADSCs) and human umbilical-cord-derived mesenchymal stem cells (hUCMSCs) emerged as preferred categories over bone-marrow-derived MSCs, because they can be obtained in larger volume with a significantly less invasive method [50,51]. The therapeutic benefit of MSC treatment lies in the secretome, which is a collection of signaling molecules that can alter local tissue activity at the site of injection [52]. Various factors and chemotaxic agents in the MSC secretome can promote vascularization, reduce apoptotic activity, and push tissue-specific precursor cells into differentiation [52]. Although promising, the MSC secretome does pose a risk for tumorigenesis and must be further investigated prior to being fully implemented in clinical practice [53]. It is important to note that stem cell survival is complicated, and several implantation studies may experience rejection by the host, and excessive cytotoxicity in the lesion site with poor environment that promote survival. Cytoskeleton remodeling and a lack of growth factors also limits axonal growth. Chondroitin sulfate proteoglycan degradation has been used by some studies to facilitate growth [54]. Figure 4 depicts the origins of several commonly used stem cell therapies in clinical trials.

#### 1.3.1. Stem Cells and Neuroplasticity in the Animal Model

Modern experimental therapies have focused on exploring the regenerative potential of axons further with stem cell transplantation. This raises the question of whether there may be a similar synergistic relationship between stem cell therapy and neuromodulation in SCI repair. Mu et al. 2024 explored this theory with a murine model of spinal cord compression at T10 [52]. In this study, a combination of mouse neural cortical stem cells (NSCs) and hUMSCs were used. The hUMSCs allowed for better proliferation of progenitor murine NSCs [3]. Mu et al. discovered that combined therapy with ES and NSC/hUMSC injection showed greater paw standing when compared to either treatment in isolation or the control [52]. However, limited coordinated gait was observed. TsT is believed to facilitate the fine-tuning of newly regenerated circuitry. The addition of simultaneous stepping training to this experimental model could show improvements in coordinated gait. This approach was adopted in a number of human studies that are listed in Table 3. Another line of stem cell research may include the use of Schwann cell transplantation, which successfully bridged to human clinical trials. Schwann cells are peripheral nervous system cells which can regenerate and myelinate axons [57]. Autologous Schwann cell transplantation into the spinal cord has been shown to have a regenerative and neuroprotective effect in animal models of SCI [58,59]. Human autologous Schwann cell transplantation has yet to show clinically remarkable changes in persons with SCI; however, they have been found to be safe in Phase 1 clinical trials [60]. Combination therapy, such as upregulation of cyclic AMP or administration of chondroitinase, along with Schwann cell transplantation, can expand the area of axonal regeneration past the point of cell transplantation, potentially augmenting SCI recovery [60,61].

#### 1.3.2. Stem Cells and Human Model with SCI

Marc Tuszynski’s research group has extensively demonstrated that the implantation of homologous neural stem cells in the spinal cord promotes robust corticospinal regrowth [66]. This work provided one of the first clear demonstrations that developmentally matched neural grafts can reconstitute a permissive spinal substrate long-distance corticospinal regeneration [66]. Their subsequent work has explored the mechanisms underlying regeneration and investigated how pharmacological agents can induce a regenerative phenotype in injured axons [67,68,69]. However, these regenerative advances also highlight that axonal growth and graft integration occur with many critical co-determinants, such as neuromodulation and TsT, that lead to functional recovery [67,68,69]. Clinical trials of stem cell therapies across individuals with SCI consistently show measurable improvements leading toward recovery (Table 3). MSCs were the primary type of stem cell used in human clinical trials and were associated with notable improvements in functional outcomes and synaptic connections [70]. When compared to induced pluripotent stem cell therapy (IPSCs), MSCs offer less structural support [60]. Although the IPSCs offer more robust regenerative outcomes, they are more restrained by safety regulations and remain early in clinical trials [71,72]. The findings show that stem cells are a promising intervention to recovery but are not stand-alone treatment methods. Levi et al. suggests that neural stem cell implantation alone is not enough to drive meaningful recovery in SCI [62]. The trial showed that alone modest gains were made, and a combinational approach would be required to allow functional plasticity [62]. It is worth noting that most of the stem cells studies have encouraged the applicants to engage in TsT to promote motor recovery [63,64,65]. Stem cell therapy has also been used in demyelinated models similar to amyotrophic lateral sclerosis and chronic stroke [73,74,75]. The early feasibility trials indicated the safety of administering stem cells as well as tolerance of the therapy by all participants [73,74,75].

A cause of concern in the use of stem cells is in the delivery system (Figure 4). Recent studies have emphasized the need to develop an effective vehicle to integrate stem cells with the host cells after implantation [51]. Another concern is the existence of extrinsic [glial scar and inhibitor Nogo molecules] and intrinsic barriers [cytokines, diminished transcription factors, lack of growth factors] for axonal regeneration [76]. Du et al. highlighted the use of electrical stimulation combined with conductive biomaterial scaffolds to provide the structural and bioelectrical cues to promote growth [51]. In rodent models, neural stem cells delivered with the biomaterial scaffolds exhibited greater survival, differentiation into different neuronal subtypes, and promoted axonal regeneration when compared to stem cells without scaffolding [66]. Together, these results illustrate how stem cell delivery with the appropriate strategies can be used to promote recovery after SCI.

## 2. Summary and Conclusions

This perspective article highlights the use of neuromodulation, task-specific training, and stem cell therapy as a potential synergistic pathway for recovery of volitional sensorimotor control in persons with SCI. Stem cell therapy may serve as a future regenerative venue for spinal cord injury. However, there is currently limited clinical evidence about its effectiveness in persons with SCI. There is a growing body of knowledge surrounding treatment for SCI with motor function loss. Many attempts at regenerating damaged neural circuits have been accomplished with applications of SCES, physical rehabilitation, and stem cell therapy, both in isolation and in pairs with each other. Neuromodulation alone can offer partial recovery of volitional movement; however, TsT in isolation has shown minimal success, especially in persons with complete or higher level of SCI. It is becoming increasingly clear that there is a synergism between neuromodulation and TsT. Neuromodulation alone can promote recovery of lost neural circuitry, while simultaneous TsT reorganizes those newly regenerated circuits to allow for fine motor control. On the other hand, stem cells have been used in clinical trials for SCI recovery. The emphasis of work relies on the secretions of growth factors and cytokines at the injection site to promote vascularization, reduce apoptosis, and modulate inflammation of the damaged axons. In summary, neuromodulation and TsT primarily enhance spared circuits and promote compensatory sprouting, whereas stem cell therapy restores connectivity through axonal repair across the legion [14,63]. Together these are complementary, because stem cells regenerate the structural pathway while neuromodulation and TsT are strengthening and refining the pathway. The advent of clinical trials utilizing stem cell therapy raises the question of whether combining the three approaches may result in even greater recovery in persons with SCI.

There are limitations associated with existing research in the field, notably that most murine studies incorporate stem cell therapy in the acute phase of SCI. This is not necessarily generalized with most humans with SCI who receive treatments in more chronic stages of disease [3]. As cited by Mu et al. and Shang et al., highlighting the significance of timeliness with the administration of stem cell therapy [50,77], one study found that NSC therapy in the subacute phase generated a greater response than in the chronic phases [78]. It is important to note that the secretome of the MSCs containing growth factors like vascular endothelial growth factor (VEGF) may promote angiogenesis. This does pose a risk for tumorigenesis, which must be investigated further prior to larger clinical trials in persons with SCI [53]. Additionally, numerous factors can impact neuroplasticity, such as the timing of therapy relative to the injury and the behavioral context in which neuromodulation is applied. For example, typical functional electric stimulation or transcranial magnetic stimulation-based approaches rely on spike-timing-dependent plasticity and are tightly paired with movements. When neuromodulation is delivered alone it is ineffective, but when paired with TsT, it enhances neuroplasticity [79]. Administering therapy in the chronic phase of injury may not be as effective [79]. The dose of therapy may also vary depending on injury severity and can differentially affect neuroplasticity [80].

In conclusion, these findings highlight the unique contributions of each strategy for improving the quality of life in a person impacted by SCI and explain the rationale of prospectively combining these rehabilitation strategies together. Future studies should aim to take advantage of the synergistic relationships of existing experimental therapies.

## Figures and Tables

**Table 1:** Representative studies of Neuromodulation Therapy for Restoring Motor Control in Animal Models.

Study	Neuromodulation	Task-Specific Training	Measurements	Key Findings
Musienko et al., 2011 [20]	Lumbosacral EES + pharmacologic tuning	Stepping training	Electromyography	1. Control of dopaminergic, noradrenergic, and serotonergic receptors using pharmacotherapy allowed for fine tuning of hind-limb movements in rats with SCI 2. Combination of EES with monoamine pharmacologic tuning can synergistically upregulate movement in rats with SCI.
Bonizzato et al., 2018 [24]	Lumbosacral EES + Deep Brain Stimulation (open- and close-looped) + pharmacologic tuning	Stepping Training	Behavioral Assessments	1. The addition of deep brain stimulation with serotonergic agonists and EES results in clear improvement after SCI. 2. Locomotor performance differed between stimulus conditions.
Zareen et al., 2018 [31]	Motor cortex stimulation	N/A	Stereological quantification using optical fractionator	Measurable increase in biochemical pathways for axonal growth and formation of new synapses, mTOR and Jak/Stat, respectively, following motor cortex stimulation.
Asboth et al., 2018 [23]	Lumbosacral EES + pharmacologic tuning	Stepping training	Behavioral Assessments, Anterograde Tract Tracing	Recovery after sever SCI was driven cortico-reticulo-spinal reorganization rather than corticospinal regeneration enabled by spinal neuromodulation.
Sharif et al., 2021 [8]	Dual trans-spinal direct stimulation and motor cortex intermittent theta burst stimulation	Horizontal ladder training	CST anterograde tracing, Video analysis of forelimb placement/precision	1. Rats with SCI who underwent combined physical training and neuromodulation had a statistically significant (*p* < 0.05) increase in regained motor function at six weeks of rehabilitation when compared to rats that were treated with rehabilitation alone. 2. There was a statistically significant (*p* < 0.04) increase in corticospinal tract axonal sprouting caudally in the neuromodulation + task specific training group versus the rats who underwent training alone
Williams et al., 2024 [47]	Motor cortex and trans-spinal direct stimulation with healing biomaterial (C_fphs_)	N/A	Immunohistology, Fluorescence microscopy	C_fphs_ administration 3 days following SCI reduced proliferation of secondary and tertiary stage pathology, creating a better environment for subsequent neuromodulation therapy. Significant enhancement in corticospinal tract axon density was observed in injured rat models when compared to injured rats that received C_fphs_ alone.

N/A: not applicable.

**Table 2:** Representative studies of Neuromodulation therapy for restoration of motor control in the human model.

Study	Neuromodulation	Task-Specific Training	Measurements	Key Findings
Harkema et al., 2011 [14]	Lumbosacral EES	Stepping training on a treadmill and standing training	Electromyography, footswitch, ground reaction forces, joint angles, body weight support	1. EES during task-specific training showed significant EMG activity when compared to task-specific training without EES. 2. A patient with SCI could perform voluntary dorsiflexion at the ankle, flexion at the knee, and toe extension on verbal command with epidural stimulation while supine following 80 sessions of stand-training.
Grahn et al., 2017 [32]	Lumbosacral EES	Side-lying lower extremity flexion/extension, steplike movement in upright position, standing training	Electromyography	A patient with SCI was able to stand without trainer assistance for longer than 1.5 min. Patient was also able to voluntarily create step-like movements while upright as well as while side-lying with constant EES.
Rejc et al., 2017 [33]	Lumbosacral Epidural SCS	Long-term activity-training (standing, stepping, volitional movements)	EMG, motion capture, ground reaction forces, volitional movement attempts, coordination analysis (JPD)	A patient with chronic motor complete SCI regained volitional lower limb movement and independent standing
Angeli et al., 2018 [35]	Lumbosacral EES	Standing and Step Training	EMG	Two participants with complete SCI achieve volitional control of lower-limb muscles and walking with assistive devices
Gill et al., 2018 [28]	Lumbosacral EES	Locomotor and stand training	EMG	Neuromodulation of lumbosacral networks enabled independent stepping and standing while TST reinforced network plasticity.
Wagner et al., 2018 [29]	Targeted Spatiotemporal Neuromodulation	Gait Training and Walking	EMG	Three participants with chronic SCI achieved stepping and balance assisted ambulation
Kazim et al., 2021 [34]	Epidural and Transcutaneous Stimulation, trans-spinal magnetic stimulation	Treadmill, skilled task training	CST Sprouting, EMG, MEPs, DTI imaging	Neuromodulation and task-specific training synergistically enhanced plasticity in corticospinal circuits, evidence of reorganization at all levels of the motor system
Samejima et al., 2022 [48]	Epidural and Transcutaneous stimulation Open-loop and Closed-loop	Reach/grip tasks, exoskeleton-assisted training	EMG, Motor evoked potentials (MEPs), Functional assessments, H-reflex Modulation	Spinal Cord Stimulation (SCS) promotes activity-dependent plasticity and motor recovery, Closed-loop may offer enhanced neuroplasticity
Mckenzie et al., 2024 [16]	Transcutaneous spinal cord stimulation, acute intermittent hypoxia	Gait training	10 m walk test, 6 min walk test, Timed Up and Go test (TUG)	Combination of neuromodulatory therapies (AIH, gait training, and tSCS) showed significant improvement in chronic SCI patient’s ability to perform the TUG test when compared to those with tSCS and training alone as well as just training.
Comino-Suarez et al., 2025 [30]	Transcutaneous spinal cord stimulation	Robotically assisted walking (Lokomat)	Lower Extremity Motor Score (LEMS), dynamometry, Electromyography,10 m walk test, 6 min walk test, Timed Up and Go test	Controlled clinical study that showed improvement in lower-extremity motor function and the ability to walk in patients with subacute SCI following combined tSCS and robotic-assisted walking training when compared to those who received sham tSCS stimulation.

**Table 3:** Representative studies of administering stem cells in the human model.

Study	Type of Stem Cells	Supportive Therapy	Measurements	Key Findings
Levi et al., 2018 [62]	NSCs	Rehab	ASIA scores	Limited functional improvement as a standalone treatment
Xia et al., 2023 [63]	MSCs	Rehab + Pharmacological support	ASIA scores, functional outcomes	Improved neuroplasticity and motor recovery
Bhatt & Das, 2025 [64]	MSCs and IPSC	Rehab + Pharmacological support	ASIA scores, EMG, fMRI	Functional recovery, evidence of CST reorganization and plasticity
Gartit et al., 2025 [65]	MSCs	Rehab	ASIA scores	Long-term neuroplastic changes and greater functional recovery

## Data Availability

No new data were created or analyzed in this study. Data sharing is not applicable to this article.

## Abbreviations

CNS	central nervous system
CST	corticospinal tract
EAW	exoskeletal assisted walking
hUMSC	human umbilical Mesenchymal stem cell
IPSCs	induced pluripotent stem cell therapy
MSC	Mesenchymal stem cell
NSC	neural stem cell
PA	proprioceptive afferent
SCI	spinal cord injury
SCES	spinal cord epidural stimulation
tSCS	transcutaneous spinal cord stimulation
TsT	task-specific training
